# Supplementary material for: Manganese Deficiency Suppresses Growth and Photosynthetic Processes but Causes an Increase in the Expression of Photosynthetic Genes in Scots Pine Seedlings
Source: Cells. 2022 Nov 28;11(23):3814. doi: 10.3390/cells11233814 (PMC9739257; doi:10.3390/cells11233814)
Supplement: Supplementary file 1 [file cells-11-03814-s001.zip › cells-2060347-supplementary.pdf]

Table S1. List of primers used for qRT-PCR analysis

№	Gene Bank ID	Gene	Description	Primer 5′-3′	
1	Z16409.1	*lhca*	Lhca4 protein, Type 4 protein of light-harvesting complex of photosystem I	CCAGCATTGGCCTGATCAAC	CCACTTCGTTGGGAGGAAGA
2	AAB19040.1	*lhcb2*	Light-harvesting chlorophyll a/b-binding polypeptide, chloroplastic	TGCCAAGAACAGAGAGCTGG	ATGCTCTGGGCGTGAATCAA
3	AET45557.1	*psaA*	Photosystem I P700 chlorophyll a apoprotein A1	GGGAGGTGGTGATTTGGTGA	AAGCGAAAACCCAGATTCGC
4	ALK01273.1	*psaB*	Photosystem I P700 chlorophyll a apoprotein A2	TGTCAGGACTCTTCGGAGTCAG	GACCTGTGAAAAGCGGTCCT
5	ABO77179.1	*psbA*	Photosystem II protein D1	TGAAGGTTACAGATTCGGTCA	TGAATATGCAACAGCAATCCA
6	AET45537.1	*psbB*	Photosystem II CP47 reaction center protein	GGAGTGGGTGGAACATCACC	ACACCCATATCCCAGGACCA
7	AET45561.1	*psbC*	Photosystem II CP43 reaction center protein	CGACTCTTAACCCAAGTGCT	GCTTCTCCGGACCATACGAA
8	ADX78157.1	*psbD*	Photosystem II protein D2	GATATTATGGATGACTGGTTACG	CATTCTCTGGTCCCATTGC
9	AAD28778.1	*psbS*	Photosystem II 22 kDa protein, chloroplastic non-photochemical quenching	CCTATATACGAGGCAGAGC	ACTCCCGTCTCAATGTTGA
10	AET45528.1	*petA*	Cytochrome *f*	GATAAAGAAGCTCACTTCCTG	CCACCGTCTGATGTATTATCG
11	AET45542.1	*petD*	Cytochrome b_6f complex subunit 4	CTATCGTCCGACCGTTACAG	GAATCCACCTGTGAGATAAACAC
12	BT109679.1	*petC*	Cytochrome C1	CGGATCTGGAGGTTCTGGA	CCATCATTCTCGACAACAAGA
13	EF081988.1	*petE*	Plastocyanin	TTCTGATGGGTGCAGCA	CGGCGTTGTTCTTGAAG
14	AET45533.1	*rbcL*	RuBisCO large subunit	CAACCATTCATGCGCTGGAG	TGCGGTAAAACCTCCCGTC
15	A0A0B5KFY1*	*psbO*	Photosystem II manganese-stabilizing polypeptide	CAACCAAGATCGCAGCAGTC	TTCCAGGTACGTCTGGCTCT
16	PITA_000005150 **	*psbP*	PsbP protein 1, chloroplastic	GAGTAGCAGCCCTAACCAGC	GCTGGGGTTCCATTTTGACG
17	A9NZN7*	*psbQ*	Oxygen-evolving enhancer protein 3-2, chloroplastic	AGAGCGGACACATGATGAGC	ACTGATAGGACCCAGGGCTT
18	O22599	*porA*	NADPH-protochlorophyllide oxidoreductase PORA	ACTACTGGACTCTTCCGCGA	GTGCAAGCCTTTTTCCTGCT
19	Q00864	*chlB*	Light-independent protochlorophyllide reductase subunit B	GTGGAGTTATTTCCGCCCCA	TCCTCCATACCCAGAGCGAA
20	G8J3Y5*	*chlN*	Light-independent protochlorophyllide reductase subunit N	AGAACCCCGCTATGCAATGG	TGATGACGCTGGGGTCTCTA
21	P41645	*chlL*	Light-independent protochlorophyllide reductase iron-sulfur ATP-binding protein, subunit L	CCGGGTGTGGAGGATATGTG	AAACCTCCACAGACCACGTC
22	AAX92681	*Actin*	Actin 1	TTAGCAACTGGGATGACATGGA	CCTGAATGGCAACATACATAGCA

* - UniProt IDs (https://www.uniprot.org); ** - ConGenIE.org IDs (https://plantgenie.org/)

Table S2. The contents of nutrients in the roots of Scots pine seedlings

Nutrient	Control					Mn-deficient				
	4th week	6th week	12th week	19th week	24th week	4th week	6th week	12th week	19th week	24th week
K	ND	ND	359.7±27.0	407.5±13.7	369.0±27.5	ND	ND	480.3±21.5*	417.5±30.0	492.7±36.5*
Mg	39.5±1.1	33.6±1.0	26.3±0.4	13.9±0.9	35.9±3.5	41.7±0.9	33.4±1.2	21.8±0.3*	18.0±1.8	35.7±1.3
Ca	ND	ND	7.69±0.55	8.49±0.52	13.47±0.67	ND	ND	7.94±1.01	12.11±1.21*	13.45±0.80
P	ND	ND	218.0±2.0	197.9±4.7	157.0±10.2	ND	ND	204.5±13.3	210.8±17.1	172.1±12.5
Mn	1.55±0.25	3.17±0.34	0.95±0.29	0.57±0.03	0.99±0.07	0.18±0.00*	0.04±0.01$^{\times}$	0.06±0.01*	0.10±0.01$^{\times}$	0.10±0.00$^{\times}$
Fe	164.1±12.0	181.4±18.9	ND	ND	172.9±6.2	184.3±12.6	153.8±9.9	ND	ND	183.9±16.7
Zn	2.17±0.20	2.07±0.09	2.00±0.12	2.62±0.24	2.07±0.07	2.67±0.10	3.28±0.43*	2.55±0.04*	3.17±0.65	4.29±0.23$^{\times}$
Cu	ND	ND	235.7±6.3	230.6±10.9	353.6±52.8	ND	ND	249.2±3.2	239.5±8.5	268.8±14.8

ND – no data. Pairwise comparisons of the means were performed between control and Mn-deficient plants at each separate time point using Student's t test for normally distributed data or the Mann–Whitney rank sum test when the t test was not applicable. Asterisks (*, for Student's t test) or multiplication signs (×, for Mann–Whitney rank sum test) denote significant differences at $p < 0.05$.

Table S3. The contents of nutrients in the needles of Scots pine seedlings

Nutrient	Control						Mn-deficient					
	4th week	6th week	12th week	19th week	24th week	24th week upper needles	4th week	6th week	12th week	19th week	24th week	24th week upper needles
K	ND	ND	258.1±74.5	295.5±14.1	329.9±26.7	349.2±10.8	ND	ND	472.0±22.1 *	401.3±8.0 *	409.0±42.8	523.1±36.8 ×
Mg	43.5±0.9	46.4±1.1	43.2±1.2	72.2±3.5	63.8±4.8	51.2±1.6	45.9±0.4	44.4±1.6	46.9±1.4	70.7±4.0	85.7±6.2*	65.4±3.0*
Ca	ND	ND	47.8±7.2	58.6 ±4.1	71.1±3.4	50.7±2.5	ND	ND	60.7±8.2	55.8±4.1	92.2±7.9*	59.8±5.9
P	ND	ND	121.3±4.9	109.9±4.5	92.1±5.1	74.2±2.7	ND	ND	123.7±5.5	112.9±3.4	101.3±7.8	84.1±3.7×
Mn	3.65±0.19	5.75±0.21	6.50±0.32	6.18±0.63	5.92±0.55	4.78±0.28	0.61±0.12 *	0.34±0.03 ×	0.21±0.02 *	0.11±0.01 ×	0.10±0.02 ×	0.05±0.01 ×
Fe	1.44±0.06	1.49±0.03	1.95±0.09	1.80±0.12	1.20±0.12	0.92±0.08	1.25±0.15	1.42±0.06	1.73±0.20	1.55±0.12	1.22±0.08	1.20±0.11 ×
Zn	1.12±0.05	0.97±0.04	0.85±0.03	1.13±0.08	1.00±0.12	0.70±0.05	1.19±0.03	1.06±0.02	1.01±0.02 *	1.18±0.08	1.21±0.10	0.79±0.03
Cu	ND	ND	148.6±3.8	154.5±6.1	126.2±8.4	133.6±7.1	ND	ND	143.9±6.7	151.7±6.2	134.7±4.2	134.2±7.7

ND – no data. Pairwise comparisons of the means were performed between control and Mn-deficient plants at each separate time point using Student's t test for normally distributed data or the Mann–Whitney rank sum test when the t test was not applicable. Asterisks (*, for Student's t test) or multiplication signs (×, for Mann–Whitney rank sum test) denote significant differences at $p < 0.05$.

Table S4. The transcript levels of photosynthetic genes (*Lhcb2, psbA, psbB, psbC, psbD, psbO, psbP, psbQ, psbS, petA, petC, petD, petE, Lhca, psaA, psaB* and *rbcL*), and genes involved in chlorophyll biosynthesis (*porA, chlB, chlL, chlN*) in the needles of Scots pine seedlings

Genes	Control			Mn-deficient		
	6th week	19th week	24th week	6th week	19th week	24th week
Lhcb2	1.00 ± 0.00	4.26 ± 0.33	1.67 ± 0.10	1.49 ± 0.10×	3.28 ± 0.24*	3.65 ± 0.27×
psbA	1.00 ± 0.00	2.79 ± 0.15	2.67 ± 0.11	0.82 ± 0.08	3.27 ± 0.19	4.11 ± 0.13*
psbB	1.00 ± 0.00	2.67 ± 0.11	2.01 ± 0.05	1.10 ± 0.09	2.59 ± 0.05	3.45 ± 0.15×
psbC	1.00 ± 0.00	3.43 ± 0.26	1.69 ± 0.09	0.94 ± 0.06	2.51 ± 0.15*	3.31 ± 0.14*
psbD	1.00 ± 0.00	2.92 ± 0.27	1.23 ± 0.05	0.57 ± 0.07×	0.91 ± 0.13*	2.59 ± 0.20×
psbO	1.00 ± 0.00	3.80 ± 0.11	1.36 ± 0.05	1.34 ± 0.06×	3.45 ± 0.28	3.14 ± 0.14×
psbP	1.00 ± 0.00	3.10 ± 0.23	1.78 ± 0.09	1.13 ± 0.07	2.07 ± 0.14*	2.87 ± 0.24*
psbQ	1.00 ± 0.00	3.17 ± 0.08	1.37 ± 0.05	1.37 ± 0.03×	2.71 ± 0.14*	2.84 ± 0.13*
psbS	1.00 ± 0.00	2.79 ± 0.16	2.90 ± 0.16	1.40 ± 0.06×	2.80 ± 0.20	4.87 ± 0.22*
petA	1.00 ± 0.00	3.54 ± 0.10	1.46 ± 0.13	1.87 ± 0.05×	3.39 ± 0.26*	3.89 ± 0.25*
petC	1.00 ± 0.00	2.49 ± 0.11	2.85 ± 0.12	1.10 ± 0.02×	3.31 ± 0.18×	4.00 ± 0.14*
petD	1.00 ± 0.00	3.84 ± 0.11	1.51 ± 0.08	1.42 ± 0.08×	2.72 ± 0.13*	3.53 ± 0.11*
petE	1.00 ± 0.00	4.02 ± 0.08	1.61 ± 0.07	1.62 ± 0.09×	2.77 ± 0.12*	2.66 ± 0.11×
Lhca	1.00 ± 0.00	3.36 ± 0.35	1.55 ± 0.03	1.17 ± 0.05	3.64 ± 0.13	4.94 ± 0.21×
psaA	1.00 ± 0.00	2.47 ± 0.20	1.80 ± 0.21	1.47 ± 0.11×	1.46 ± 0.12*	3.07 ± 0.12*
psaB	1.00 ± 0.00	2.48 ± 0.14	1.38 ± 0.13	1.54 ± 0.14×	1.46 ± 0.11*	2.54 ± 0.27×
rbcL	1.00 ± 0.00	3.96 ± 0.35	1.23 ± 0.04	1.29 ± 0.04×	1.79 ± 0.23*	3.46 ± 0.14×
porA	1.00 ± 0.00	4.58 ± 0.40	1.01 ± 0.04	1.28 ± 0.06×	3.11 ± 0.41*	1.60 ± 0.25×
chlB	1.00 ± 0.00	4.08 ± 0.42	1.14 ± 0.04	1.54 ± 0.08×	3.97 ± 0.22	3.98±0.25*
chlL	1.00 ± 0.00	3.94 ± 0.23	0.95 ± 0.04	1.55 ± 0.07×	2.61 ± 0.37*	3.49 ± 0.35×
chlN	1.00 ± 0.00	3.61 ± 0.17	0.83 ± 0.07	1.37 ± 0.07×	1.57 ± 0.12*	2.67 ± 0.30*

Pairwise comparisons of the means were performed between control and Mn-deficient plants at each separate time point using Student's t test for normally distributed data or the Mann–Whitney rank sum test when the t test was not applicable. Asterisks (*, for Student's t test) or multiplication signs (×, for Mann–Whitney rank sum test) denote significant differences at $p < 0.05$.
